# Codon Harmonization of a Kir3.1-KirBac1.3 Chimera for Structural Study Optimization

**DOI:** 10.3390/biom10030430

**Published:** 2020-03-10

**Authors:** Evan Van Aalst, Maryam Yekefallah, Anil K. Mehta, Isaac Eason, Benjamin Wylie

**Affiliations:** 1 Department of Chemistry and Biochemistry, Texas Tech University, Lubbock, TX 79423, USA; Evan.Van-Aalst@ttu.edu (E.V.A.); Maryam.Yekefallah@ttu.edu (M.Y.); Isaac.Eason@ttu.edu (I.E.); 2 National High Magnetic Field Laboratory and McKnight Brain Institute, University of Florida, Box 10015, Gainesville, FL 32610, USA; anil.mehta@ufl.edu

**Keywords:** codon harmonization, nuclear magnetic resonance (NMR), codon bias, protein folding, protein expression, K^+^ channels

## Abstract

The expression of functional, folded, and isotopically enriched membrane proteins is an enduring bottleneck for nuclear magnetic resonance (NMR) studies. Indeed, historically, protein yield optimization has been insufficient to allow NMR analysis of many complex Eukaryotic membrane proteins. However, recent work has found that manipulation of plasmid codons improves the odds of successful NMR-friendly protein production. In the last decade, numerous studies showed that matching codon usage patterns in recombinant gene sequences to those in the native sequence is positively correlated with increased protein yield. This phenomenon, dubbed codon harmonization, may be a powerful tool in optimizing recombinant expression of difficult-to-produce membrane proteins for structural studies. Here, we apply this technique to an inward rectifier K^+^ Channel (Kir) 3.1-KirBac1.3 chimera. Kir3.1 falls within the G protein-coupled inward rectifier K^+^ (GIRK) channel family, thus NMR studies may inform on the nuances of GIRK gating action in the presence and absence of its G Protein, lipid, and small molecule ligands. In our hands, harmonized plasmids increase protein yield nearly two-fold compared to the traditional ‘fully codon optimized’ construct. We then employ a fluorescence-based functional assay and solid-state NMR correlation spectroscopy to show the final protein product is folded and functional.

## 1. Introduction

Solid-state nuclear magnetic resonance (SSNMR) is a key structural technique to study membrane proteins within their native or native-like bilayer environment. Indeed, the inherent plasticity of these structures and their dependence upon their lipid environment renders them challenging targets for X-ray crystallography. Membrane protein function strongly depends upon the composition and phase of the surrounding lipids, further necessitating structural studies within the bilayer environment at physiological temperature. While many SSNMR studies quantify the structure and dynamics of membrane proteins within bilayers, the advancement of this technology is often limited by sample availability: the quantity of protein produced per gram of ^15^N, ^13^C, and/or ^2^H enriched material is a major limiting factor for NMR studies. While simpler eukaryotic expression systems such as *Pichia pastoris* show great promise in high-yield expression of isotopically enriched material, they do not share the simplicity of *E. coli* expression, and extensive selective or skip labeling strategies are still under development [1,2,3,4]. Thus, there is still much to be gained by improving the expression yield of functional proteins from prokaryotic expression systems. A major means to this end is the optimization, or codon harmonization, of plasmids. Here, we present an extensive study of codon harmonization in expression of a membrane protein from minimal media, and show we produce large quantities of functional, folded protein. As sample preparation for nuclear magnetic resonance (NMR) studies is costly, it is of vast importance to optimize all aspects of expression and purification to generate efficient protocols. In doing so, it is pertinent to consider all variables that may affect protein quality and yield. The wider use of codon harmonization in the NMR community could be another powerful tool to aid in the ever-necessary quest to increase recombinant protein expression for structural studies.

There are many variables within a gene sequence and its corresponding mRNA that can influence protein quality and yield. A 5′ mRNA rare codon ‘ramp’ may help to prevent ribosomal jamming along the mRNA during elongation by slowing initiation or function to prevent 5′ mRNA secondary structure formation [5]. The presence of Shine–Delgarno-like (SD-like) sequences within the mRNA may contribute to ribosomal stalling during elongation, as the 16S subunit can bind to it and slow the translational rate [6]. tRNA depletion due to overrepresentation of specific codons in the mRNA may also contribute to ribosomal stalling, as elongation is halted from lack of charged tRNAs [7]. mRNA secondary structure formation can inhibit both translation initiation and elongation. This takes place through two mechanisms; strong 5′ structure formations that inhibits ribosomal binding and the formation of inter-strand secondary structures that require unwinding before elongation may continue [8,9]. These variables affect translational initiation, elongation, or both and cause deconvolution of individual contributions to overall rates to be an arduous process.

There is evidence to suggest that the exclusive use of codons corresponding to the more abundant tRNAs in the expression organism may, in some cases, lead to rates of translation that exceed the time required for proper protein folding [10,11,12]. Often, this loss is acceptable; the yield of folded protein may still be high enough that further optimization is not required. This is likely true of many globular proteins which are standardly optimized for the expression system. In such cases most codons chosen will correspond to the most abundant tRNAs within the expression organism. Unfortunately, strategies for sequence optimization outside the traditional optimization are often overlooked. Codon choice among synonymous codons is not random, but the product of selective evolutionary pressure [13]. The reason for this is not definitively understood, though experimental evidence suggests a multitude of functionalities such as relieving tRNA pool depletion and selection against mRNA secondary structure [14,15]. Evidence suggests that, as a function or a byproduct, rarer codons can fine-tune the rate of translation, potentially allowing for the proper folding of proteins [15]. In heterologous expression systems, it has been previously shown that changing the relative abundance of codons used to more accurately reflect the native gene sequence can lead to differences in local translation rates that ultimately affect protein quality and yield [16,17].

Codon harmonization is the design of recombinant gene sequences using synonymous codons in the expression organism to match the codon landscape within the native organism [15,17]. It has been experimentally shown to increase recombinant protein yield, presumably through mitigation of protein misfolding, in some favorable cases [18]. The premise is simple: by substituting in rarer codons to match native codon usage, the rate of translation will be slowed at presumably key points due to corresponding tRNA limitation [19]. This in turn is hypothesized to allow time for the nascent peptide to fold the secondary structure for proper function [15]. Codon harmonization is not a new concept, though it’s use under this name has been relatively sparse over the last fifteen years [16,17,20,21,22,23]. To this end, there are a number of tools, algorithms and methodologies available for generating codon-harmonized DNA sequences [16,20,23]. Codon Wizard (CW) is one such application, and can be used for both traditional optimization and codon harmonization [23]. Several filters can be selected, such as avoiding repeat motifs, consideration of metabolic circumstances, ‘tolerance’ level for codon substitution, and Guanine:Cytosine (G:C) content optimization to a preset level.

Here, we apply codon harmonization to a previously studied *Mus musculus* inward rectifier K^+^ (Kir) channel 3.1—*Paraburkholderia xenovorans* Kirbac1.3 chimera [24]. The Kir3.1 portion of this chimera comprises the cytoplasmic domain, including the N-terminus and the entire C-terminal domain (CTD), while the KirBac1.3 segment spans the transmembrane region [24]. Thus, the channel is regulated in the same fashion as mammalian G protein-coupled inward rectifier K^+^ channels (GIRKs), also called the Kir3 family. The Kir3/GIRK family is a family of Kir channels which are expressed in the central nervous system and the brain of animals [24]. Activation of GIRK channels leads to hyperpolarization of the neuron’s membrane potential, providing a fundamental component of inhibition required for nerve cell communication in both normal and diseased states [25]. Given this, they are attractive drug targets for treating neurological disorders. GIRK channels are activated by the G protein βγ subunit (Gβγ) and small compounds like ethanol in the presence of signaling lipids, primarily phosphatidylinositol-4,5-bisphosphate (PIP2). However, there are many gaps in our understanding of the mechanistic details [26,27], which may be elucidated by SSNMR. A better understanding of the molecular mechanism underlying the activation of GIRK channels is needed. Such studies will inform the design of new drugs that specifically modulate the activity of GIRK channels. Unfortunately, expression of relevant membrane proteins such as GIRK channels are logistically restricted by protein yield.

Here we show that application of codon harmonization can increase membrane protein expression yields such that structural studies, especially isotopic labeling for NMR, become economically feasible. We designed four plasmids to compare to the fully codon-optimized chimeric K^+^ channel construct. The first, named DNA codon usage for measured base optimization (DUMB Optimization, DO), was generated via our own method (Materials and Methods). The next three were designed using Codon Wizard’s ‘no tolerance’ and ‘full tolerance’ functions. Using these plasmids, we show how applications of codon harmonization can positively influence the yield of membrane proteins for NMR and functional studies.

## 2. Materials and Methods

### 2.1. Codon Harmonization

We obtained native gene sequences from the European Nucleotide Archive (Kir3.1 BAA08079.1, KirBac1.3 ABE28710.1) and used the Graphical Codon Usage Analyzer (GCUA, www.gcua.schoedl.de) to visualize the pattern of relative codon usage (RCU) in the native genes and all constructs (Figures S1–S6) [28]. Here, ‘relative adaptiveness’ of 100% denotes the most abundant corresponding tRNA in the organism [28]. For the purposes of this study, any codon with native codon usage < 40% RCU is designated as ‘rare’. This chimera is a combination of portions of two different proteins. As such, each part was harmonized using codon usage tables from its native species. Eukaryotic Kir3.1 regions were harmonized according to native *Mus musculus* codon usage. Prokaryotic KirBac1.3 regions were harmonized using *Burkholderia xenovorans* LB400 codon usage tables. Native RCU frequencies for the Chimera DNA sequence may be seen in Figure S1. Here, each codon plotted is the native codon in its respective species with designated chimera numbering conventions. All sequences were checked against the sequence of the original crystallized Chimera to verify primary structure conservation of the protein [24].

#### 2.1.1. FO and DO Sequence Design

**Figure 1:**
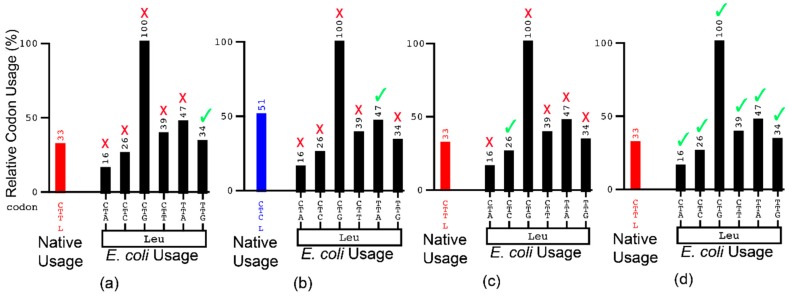
Schematic representation of harmonization methodologies for (**a**,**b**) substitution of different native codon usages for leucine according to DO methodology; (**c**) no-tolerance codon substitution; and (**d**) full-tolerance codon substitution. A green check mark designates an acceptable substitution, while a red X an unacceptable substitution. Formatting was adapted from the Graphical Codon Usage Analyzer (GCUA) output format [28].

For recombinant expression, gene sequences are typically ordered as “fully codon optimized” with the most abundantly found host codons at most or all codon positions. Here, the fully optimized (FO) construct was fully codon optimized for expression in *E. coli* through GeneArt (Thermo Fisher Scientific). RCU frequencies of the FO sequence may be seen in Figure S2. Construct codons for the DO sequence were systematically deoptimized to match the corresponding native codon abundance as closely as possible using GCUA *E. coli* usage frequencies. Codon substitution was performed such that no alternative codons in the host system were chosen with relative usage below 5% under the native usage frequency. For example, a CTT codon in the native sequence with 33% relative codon usage is substituted for TTG in the recombinant sequence with 34% relative codon usage (Figure 1a). However, a CTC codon is replaced with TTA and not CTT, as its usage is greater than 5% below CTC’s usage in *E. coli* (Figure 1b). Native codons with relative usage values below 100% were replaced with the closest E. coli codon following this logic. This serves to deoptimize each individual codon no further than the native sequence according to relative codon usage values. RCU for all codons in the DO construct may be seen in Figure S3.

#### 2.1.2. CW Sequence Design

Plasmids harmonized through CW were selected to represent the widest range of possibilities that fell within the scope of this study [23]. The use of this program involves determination of absolute differences between native and host relative codon usage frequencies. This is followed by determination of how likely a codon is to be substituted, designated the likelihood for selection to replace the donor codon (LSR). Introduction of the ‘tolerance filter’ designates which codons will appear as candidates for substitution. When ‘no tolerance’ for codon substitution is selected, the codons with the closest relative usage not exceeding the native are the only codons to appear (Figure 1c). ‘Full tolerance’ is much less specific. Here, any codon may be chosen by the CW algorithm to reflect the usage of the native sequence though ranking of LSR values favors substitution of codons with similar codon usage frequencies over more dissimilar ones (Figure 1d). The algorithm then generates a random number between 0 and 1 and begins with the codon with the highest LSR value. If the first LSR value is higher than the random number, that codon is chosen for substitution. If it is not, the LSR values of the top two codons are summed and compared to the random number. If this value is higher than the random number, then the second codon is selected for substation. If it is not, the process is repeated for the top three LSR values until a substitution is made. In this iterative approach, different sequences may be generated even when the tolerance filter is set to the same level. More detailed descriptions of the CW algorithm and methodology may be found in reference 23. The third and fourth plasmids were designed through Codon Wizard (CW) using the ‘no tolerance’ (NT) filter, designated NT and NT56, the latter is differentiated by its 56% G:C content. The fifth and final plasmid was generated via CW using the full tolerance (FT) filter, designated the FT construct.

### 2.2. Plasmid Construction

Chimera gene sequences were ordered from GeneArt and inserted into the pQE-60 expression vector using NcoI and BamHI restriction enzymes (New England Biolabs). This resulted in an N—Thrombin site—Mouse Kir3.1 intracellular—*P. xenovorans* transmembrane—Mouse Kir3.1 intracellular—Thrombin site—6x His Tag—C protein construct, as previously described [24]. The final gene sequences produced 364 residue monomer, including the C-terminal Thrombin site and His-tag, of theoretical molecular weight 40.8 kDa (163.2 kDa homotetramer, estimated with ExPASy) [29]. Gene insertion and sequence conservation were verified by sequencing.

### 2.3. Sequence Analysis and Estimation

%MinMax values for each sequence were calculated using the %MinMax input tool (www.codons.org) and visualized with Python [30]. 5′ mRNA secondary structure and minimum free energy values were predicted using RNAfold, part of the ViennaRNA WebServer [31]. Codon Adaptivity Index (CAI), Effective Number of Codons (N_c_) and Elongation Efficiency (EE) were calculated using Data Analysis in Molecular Biology and Evolution 7 (DAMBE7) [32]. Average Relative Codon Usage values were obtained by averaging GCUA output values of all codons in each construct sequence [28]. Primary amino acid sequences of Kir3.1 (UniProt, P63250), Kir3.2 (UniProt, P48542), and KirBac1.1 (UniProt, P83698) were aligned using Clustal Omega (https://www.ebi.ac.uk/Tools/msa/clustalo/) and visualized with ESPRipt 3.0 (http://espript.ibcp.fr/ESPript/ESPript/) [33,34].

### 2.4. Expression and Purification of the GIRK Chimera

M15 *E. coli* cells containing the Prep 4 plasmid were grown in M9 minimal media as previously described [35]. Briefly, 2 mM MgSO_4_, 0.1 mM CaCl_2_, 100 µg/mL ampicillin, 50 µg/mL kanamycin, 1 mL of 100x minimum essential vitamin stock, 96.22 mM Na_2_HPO_4_, 44.1 mM K_2_HPO_4_, 17.1 mM NaCl, 5 g glucose per L (0.5% *w*/*v*), 3.75 g NH_4_Cl per L (0.375% *w*/*v*), and 20 mL of solution C per L (Soln. C recipe can be find in Supplementary Information [36]) were combined and sterile filtered prior to inoculation. M9 minimal media was used for plasmid comparison to more closely match yields that would be obtained during NMR sample preparation. Three 0.5 L cultures of each construct were grown at 220 rpm, 37 °C until an OD_600_ of ~0.8 was reached. The cultures were cooled to 18 °C and induced with 0.5 mM IPTG for 24 h. Cell cultures were then pelleted at 5500 rpm for 10 min.

The pellets were resuspended in 5 mL of lysis buffer (20 mM HEPES pH 8.0, 150 mM KCl, 0.02% NaN_3_, 10 mM MgSO_4_, 250 mM sucrose) per g of cells and the following were added: 0.2 mg/mL RNase, 0.2 mg/mL lysozyme, 1 EDTA-free protease inhibitor tablet (Thermo) per 6 g cells, 1 mM benzamidine and phenylmethylsulfonyl fluoride (PMSF). Cells in the full lysis buffer were incubated at 4 °C for 30 min then lysed via passage through a homogenizer. Then, 20 mM n-dodecyl β-d-maltoside (DDM), Anatrace, Maumee, OH), 1 mM each of benzamidine and PMSF were added. The lysate was set to extract on a rocker at 4 °C for 4 h followed by ultracentrifugation at 60,000 rpm in a 70 Ti rotor (Beckman Coulter, Brea, CA) for 40 min at 4 °C to remove cell debris. The supernatant was filtered through a 0.22 μm PES bottle top filter and loaded onto a 5 mL HisTrap (GE Healthcare Life Sciences) column pre-equilibrated in wash buffer (20 mM 4-(2-hydroxyethyl)-1-piperazineethanesulfonic acid (HEPES) pH 8.0, 150 mM KCl, 10 mM imidazole, 0.02% NaN_3_, 2.5 mM DDM). The column was then treated with five column volumes of wash buffer before eluting with five column volumes of elution buffer (20 mM HEPES pH 8.0, 150 mM KCl, 250 mM imidazole, 0.02% NaN_3_, 2.5 mM DDM). Eluted protein was transferred into exchange buffer (20 mM HEPES pH 7.5, 150 mM KCl, 5 mM DDM, 2 mM dithiothreitol (DTT), 1 mM EDTA, 0.02% NaN_3_) via a HiPrep 26/10 desalting column (GE Healthcare Life Sciences) equilibrated with Exchange Buffer. The eluted protein was diluted with Exchange Buffer below 0.5 mg/mL for storage overnight. Diluted protein was then concentrated to ~3 mg/mL using an Amicon Stirred Cell with an Ultracel 100 kDa Ultrafiltration Discs (Millipore) before loading onto a HiLoad 16/600 Superdex 200 column (GEHealthcare Life Sciences) equilibrated in exchange buffer. Tetramer fractions were collected and combined.

### 2.5. Reconstitution

Lipid films were made with brain-derived phosphatidylethanolamine (PE), brain-derived phosphatidylserine (PS) and PIP_2_ in a ratio of 1:1 PE:PS with 0.5% PIP_2_ (all lipids purchased from Avanti Polar Lipids, Alabaster, AL, USA). Films were dried with N_2_ gas to remove the chloroform and put under vacuum for at least 3 h. K^+^ buffer (20 mM K-HEPES, 150 mM KCl, 1 mM EDTA, pH 7.4 w/HCl) was used to resuspend the films at a concentration of 5 mg/mL, followed by addition of 18.5 mM 3-[(3-Cholamidopropyl) dimethylammonio]-1-propanesulfonate (CHAPS, Anatrace, Maumee, OH, USA). The films were sonicated for 5–10 min until clear. Detergents were allowed to solubilize the lipids for 2 h then protein was added to a final ratio of 10:200 protein:lipid ratio. Protein was allowed to anneal for 1 h, then the first batch of Bio-Beads SM-2 Resin (Bio-Rad, Hercules, CA, USA) was added. Bio-Beads were added in equal aliquots over two to three days until samples turned cloudy, indicative of proteoliposome insolubility due to detergent removal.

### 2.6. Efflux Assay

Bio beads were filtered out using screening columns (Fisher Scientific, Hampton, NH, USA) and the proteoliposomes were mixed with 100 μM fluorescent dye 9-Amino-6-chloro-2-methoxyacridine (ACMA). Aliquots were incubated for 5–15 min, then diluted 20-fold (1:20) with Na^+^ buffer (20 mM Na-HEPES, 150 mM NaCl, 1 mM EDTA, pH 7.4 w/HCl). Dilution of the proteoliposomes serves to create a K^+^ gradient where the concentration in the proteoliposomes is much greater than outside. 20 μL of the dilute proteoliposome sample was mixed with 20 μL of Na^+^ buffer in a 384-well assay plate. Then, 8 μM carbonyl cyanide *m*-chlorophenyl hydrazone (CCCP), a H^+^ ionophore, was added immediately before starting the assay. Baseline fluorescence with CCCP was measured with excitation at 410 nm and emission at 480 nm using a Biotek synergy NEO2 fluorescent plate reader (Biotek Instruments, Winooski, VT). Ethanol was added to initiate flux and fluorescence was measured for 15 min. As K^+^ flows out of the proteoliposome CCCP transports H^+^ in, which quenches ACMA fluorescence in a dose-dependent manner. In this way, K^+^ efflux is measured as a function of H^+^ transport. 10–20 nM Valinomycin, a K^+^ ionophore, was added to measure maximal K^+^ efflux as a positive control.

### 2.7. NMR Measurements

Uniformly, ^13^C and ^15^N NMR samples were grown and purified as described in Section 2.4 using uniformly-^13^C glucose and ^15^NH_4_Cl in place of natural abundance nitrogen and carbon sources used for activity assay samples. For sample preparation, proteoliposomes were prepared as described above with several changes. First, a 1:1 protein:lipid ratio (*w*/*w*) was used, and the proteoliposomes were centrifuged at 70,000 rpm, 4 °C for 90 min (Beckman Ti70 rotor) to obtain the pellet. The pellet was resuspended in NMR buffer (20 mM HEPES pH 7.5, 50 mM KCl) and harvested at 13,000 rpm, 4 °C for 10 min in a microcentrifuge angle rotor (Eppendorf). Bulk buffer was removed followed by three to four freeze-thaw cycles with liquid N_2_ before packing into a 3.2 mm thin wall (36 μL volume) PENCIL (Agilent Technologies, Santa Clara, CA and Loveland, CO) SSNMR rotor.

The SSNMR spectrum was acquired on an 800 MHz Bruker Avance III spectrometer using a National High Magnetic Field Laboratory (Gainesville, FL) homemade low-E 3.2 mm Hydrogen-Carbon-Nitrogen (HCN) triple-resonance biosolids magic angle spinning (MAS) probe [37,38]. The sample’s spinning rate was controlled by a Bruker pneumatic MAS unit at 16.666 kHz ± 3 Hz. The spectrum was taken at a sample temperature of 233 K using ^13^C-^13^C dipolar-assisted rotational resonance (DARR) recoupling with 20 ms of mixing time. [39]. Then, 90 kHz of SPINAL-64 decoupling was applied on the ^1^H channel during the direct and indirect chemical shift evolution dimensions [40]. Non-uniform sampling of 25% was used, amounting to 87 hypercomplex points in the indirect dimension. Raw time-domain data was reconstructed using the compressed sensing algorithm and processed using TopSpin 4.0.7 (Bruker) [41,42,43]. Sine window functions, sine bell shifts of 2 and 50 Hz of line broadening in both dimensions were applied. The processed spectrum was plotted using NMRFAM-SPARKY [44].

### 2.8. Molecular Dynamics, Chemical Shift Prediction, and Torsion Angle Prediction

Molecular dynamics (MD) membrane equilibration was performed using GROMACS with inputs created in CHARMM-GUI [45,46,47]. The crystal structure (Protein Data Bank (PDB) ID: 2QKS) was downloaded from the Orientation of Proteins in Membranes database and placed into a 1:1 1-palmitoyl-2-oleoyl-phosphatidylethanolamine (POPE):1-palmitoyl-2-oleoyl-phosphatidylserine (POPS) membrane. Potassium ions contained in the crystal structure were retained to prevent the channel from collapsing. Following insertion into the membrane, the protein was equilibrated in GROMACS using the CHARMM36M forcefield and the particle mesh Ewald method (PME) was used to model electrostatic interactions [48,49]. The system was subjected to a total of 2.5 ns of equilibration MD. An average structural model was generated from the trajectories of the last 100 ps of MD. Predicted chemical shifts (at temperature and pH values corresponding to the experimental sample) for this model were generated using SHIFTX2 and then analyzed with FANDAS [50,51]. The predicted peaks were overlaid onto experimental data using NMRFAM-SPARKY. Predicted chemical shifts were used to generate backbone torsion angles for the crystal structure and the membrane-equilibrated structural model using TALOS-N [52]. Residues with significant changes in torsion angles predicted after MD relaxation were identified based on lack of overlap in comparison of dPsi and dPhi TALOS-N outputs (Supporting Information).

## 3. Results

### 3.1. Codon Harmonization Increases Recombinant Protein Yield

**Figure 2:**
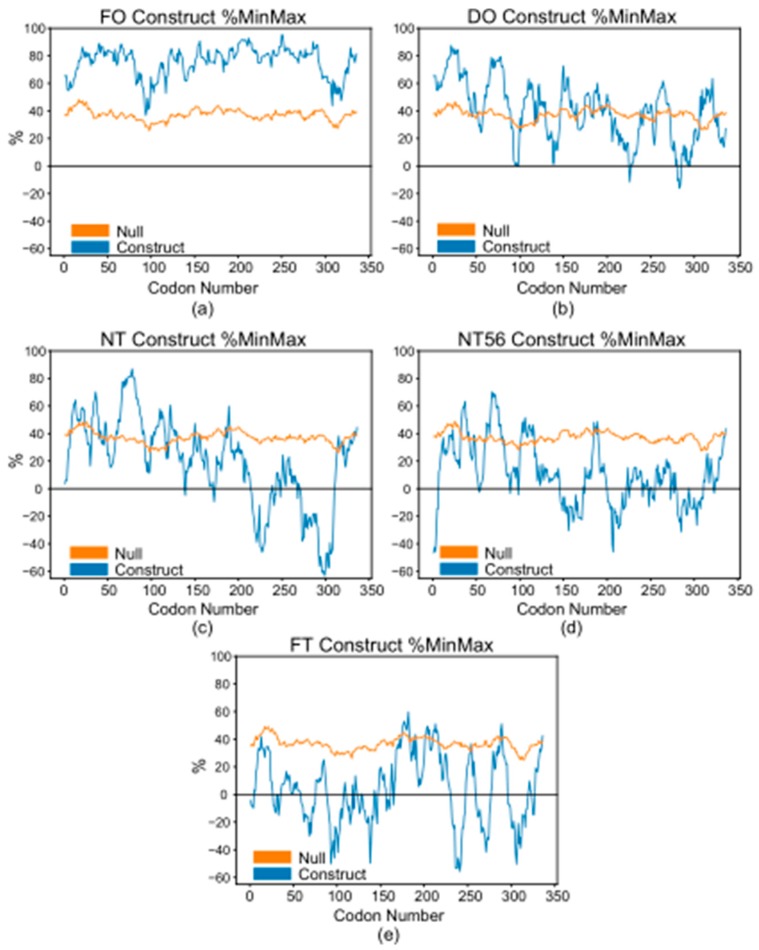
%MinMax values by codon position for (**a)** FO; (**b**) DO; (**c**) NT; (**d**) NT56; and (**e**) FT constructs. Blue indicates construct values. Orange indicates the random reverse transcription null model. Negative values (%Min) correspond to clusters of rarer codons. Positive values correspond to clusters of more optimized codons. 0% represents the average of all the possible codon choices for the construct. %MinMax values were generated with the %MinMax algorithm and plotted with Python [30].

To gauge the effect of codon harmonization on protein yield five plasmids were generated, as discussed in Materials and Methods. %MinMax values as a function of codon number are illustrated in Figure 2 [16,30]. The %MinMax algorithm accounts for a sliding window of 18 codons when calculating values. Here, +100% represents clusters of only the most abundant codons, while −100% indicates that only the rarest codons are found across the sliding window. %MinMax is a useful tool for visualizing codon usage, as it serves as an evaluation of local deoptimization as compared to a random reverse translation null model. The FO construct is the most optimized, as expected, followed by the DO construct. The NT and NT56 constructs appear to be roughly equivalent though with variation in %MinMax values at different locations and to different extents. Finally, the FT appears to be the most deoptimized of the sequences, and contains the greatest proportion of rare codons. Of note, the FT construct is the only one to contain large, sustained negative values within the prokaryotic transmembrane region (position ~70–~160) (Figure 2e).

**Figure 3:**
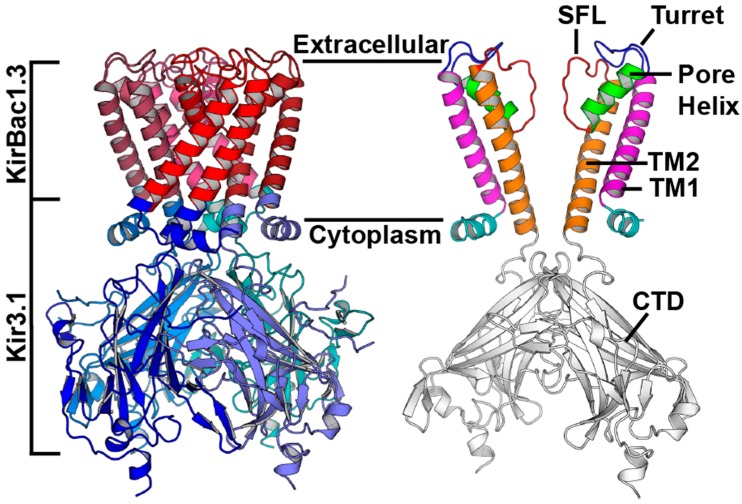
Anatomy of the chimera, defining prokaryotic and eukaryotic regions as well as transmembrane helix 1 (TM1), the turret, the pore helix, the selectivity filter loop (SFL), TM2, and the C-terminal domain (CTD). Prepared using the crystal structure (PDB ID: 2QKS) in PyMol [24].

Full graphical analysis of each construct using GCUA RCU outputs are provided in Supplementary Information, where individual codon usage values per codon are depicted (Figures S1–S6) [28]. The site-specific relative codon frequencies for each construct are mapped onto the chimera monomer (PDB ID: 2QKS) are presented in Supplementary Information Figure S7. The anatomy of the protein construct and structural regions of interest are illustrated in Figure 3. The transmembrane-extracellular region within this Chimera (residues 45–124) was harmonized to mimic the native codon usage of the corresponding residues within KirBac1.3. This was applied using codon usage tables from *Burkholderia xenovorans* strain LB400, the same strain the KirBac1.3 DNA was originally isolated from [24]. Similarly, the CTD (residues 1–44, 125–311) was harmonized using *Mus musculus* codon usage tables to more closely resemble codon usage patterns in Mouse Kir3.1. Listed residues follow the crystal structure residue numbering convention (PDB ID: 2QKS).

**Figure 4:**
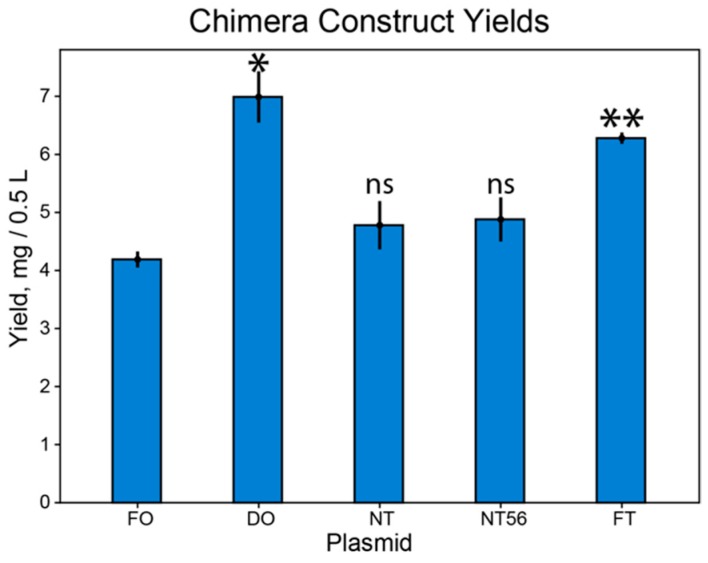
Tetrameric protein yield by construct. Statistical significance was compared against the FO construct yield. * denotes *p* < 0.05, ** denotes *p* < 0.01 in the student t test, ‘ns’ denotes no statistical significance. Error bars indicate the margin of error for three replicates.

Three 0.5 L cultures of M9 minimal media per construct were grown and purified identically. 0.5 L culture volumes were chosen to avoid losing sample due to overloading the Ni^2+^ affinity column. In this two-step purification scheme, yields of the pooled tetrameric fractions after size-exclusion chromatography were compared (Figure 4). The construct yields for the codon harmonized plasmids were greater than the more traditional FO plasmid, though not equally so and not statistically significantly in all cases. Surprisingly, the DO seemed to generate the most protein, followed closely by the FT. The NT and NT56 plasmids were comparable to the FO construct, though whether this was due solely to harmonization patterns or other variables can be debated.

### 3.2. Observed Yield Increase is Not Equal

In an effort to understand the inequality between harmonized plasmids, we turned to sequence analysis in an effort to linearly correlate some parameter to construct yield. A first guess would suggest that the extent of harmonization would play a role. In the case of the CW plasmids, this relationship seems to hold true. However, the DO construct is an outlier: of the codon harmonized sequences, the DO sequence is the least deoptimized, dictated by more positive %MinMax values (Figure 2b). Following a linear correlation between extent of harmonization and construct yield, the DO sequence should produce the lowest yield, but it does not (Figure 4). Why would this be the case?

A number of parameters were investigated to ascertain whether such a relationship could be found (Table S1). Codon adaptation index (CAI) is a measure of the extent of deviation from a set of highly expressed reference genes [53]. CAI was calculated using DAMBE7′s *E. coli* reference genes [32]. Effective number of codons (N_c_) measure bias in codon usage, where each synonymous codon is considered independent regardless of which amino acid it codes for [54]. In this way, N_c_ values range from 20 to 61. Additionally, elongation efficiency (EE) was predicted and Average Relative Codon Usage (ARCU) was calculated per construct. Of the listed parameters, one would assume EE or ARCU would be the most likely candidates for sequence characteristics linearly correlated with construct yield, given the nature of codon harmonization.

Though a trend is apparent when plotted against construct yield, it is clear that these basic characteristics are not linearly correlated with yield, suggesting functional interplay between sequence parameters (Figure S8). We find that the most likely answer to this question is that there is no one predominate factor that can be correlated to the observed experimental data. Such a system is so inundated with variables that are likely so closely tied to each other that changing one may influence any number of others. In short, we dare not conclude that a single factor is responsible for the predominant increase in protein yield. Instead, we provide a likely candidate in conjunction with patterns of harmonization that may explain the experimental trends thus identified.

### 3.3. 5′ mRNA Secondary Structure as a Potential Contributing Factor in Observed Yield Inequality

**Figure 5:**
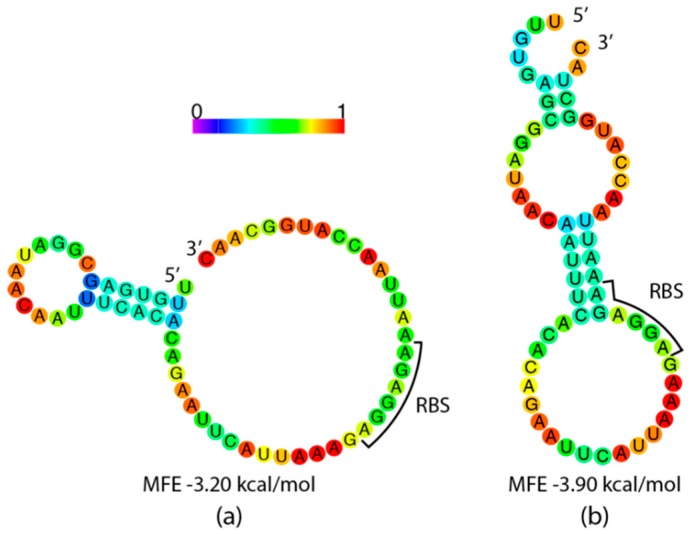
Minimum free energy structure predictions for: (**a**) FO, DO, and FT constructs; (**b**) NT and NT56 Constructs. Coloring denotes probability a given nucleotide will be involved in a base pair, with 1 denoting no predicted probability. Brackets denote the ribosomal binding site (RBS, 5′-AGGAGA-3′) encoded by the pQE-60 vector. Predicted minimum free energy (MFE) values suggest structure stability. Structures were generated using the RNAfold WebServer. [31].

Different translation initiation rates are predicted; therefore, it seems likely that initiation involves the 5′ end of the coding sequence as the sequences are identical up to the start codons. It has been experimentally determined that the ribosomal footprint extends into the coding region of an mRNA. One such study by Borujeni et al. showed this extension is 13 nucleotides (nt) long, or 16 nt beyond the ribosomal binding site in their vector [55]. Assuming this trend holds true across different vectors, we estimate that the ribosomal footprint extends through the nt at the +8 position in our chimera mRNA. This estimate (−53 through +8) was used as a basis to predict the minimum free energy (MFE) mRNA 5′ secondary structures for each construct (Figure 5). These structures represent the lowest energy conformations predicted, dictated by the listed MFE values.

The FO, DO and FT constructs are predicted to have structure-free RBSs (Figure 5a). Conversely, the NT and NT56 mRNA are both predicted to have 5′ mRNA secondary structure that occludes the ribosomal binding site (RBS) (Figure 5b). The latter two also produced the lowest yields of the harmonized plasmids. As the formation of N-terminal mRNA hairpins is known to inhibit translation initiation, this may not be a coincidence [55].

### 3.4. The Chimera is Biologically Active

A K^+^ efflux assay, first described by Su, et al., was employed with several modifications (Materials and Methods) [56]. It was paramount to ensure all constructs were properly folded into the biologically active, relevant conformations and to compare activity across constructs. Evidence suggests that deviations in codon usage from that of the native sequence can influence protein functionality [57,58]. This assay was performed first to confirm biological activity of the channel, and second to investigate activity differences across constructs.

**Figure 6:**
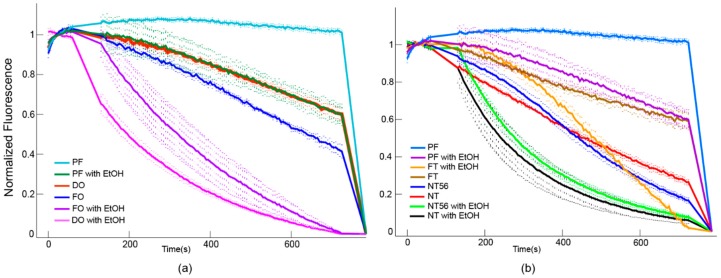
Fluorescent K^+^ flux coupled assay of (**a**) FO and DO constructs and (**b**) NT, NT56, and FT constructs using protein free (PF) liposomes with and without ethanol (EtOH) as negatives controls. Liposome composition for all samples was 1:1 PE:PS with 0.5% PIP_2_. Loss of fluorescent signal corresponds to K^+^ flux, showing all constructs are functionally active. Maximum flux is determined through addition of valinomycin.

Typically, gating of GIRK channels requires both PIP_2_ and a gating ligand such as Gβγ or ethanol to initiate K^+^ flux [26,27]. Assay data reveals that all constructs are functionally active, though slightly active in the presence of PIP_2_ only (Figure 6). MD simulations of Kir3.1 suggest an intermediate-activated state triggered by transient PIP_2_ interactions in the absence of a second gating partner that may serve to explain this observation [59]. It may be that exchanging codons at specific sites could additionally influence this channel’s basal activity, though whether or not the harmonization process contributes to this phenomenon is unknown. Here, the DO, NT, and NT56 constructs appear to be more active than the FO. As proper folding is related to function, it seems probable that harmonization processes can therefore influence both a higher quantity *and* quality.

### 3.5. SSNMR Confirms Folded Protein in the Lipid Bilayer

**Figure 7:**
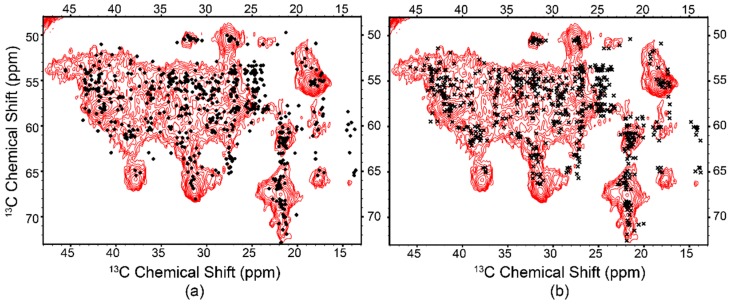
Cα-Aliphatic region of the ^13^C-^13^C DARR correlation spectrum of the GIRK chimera reconstituted into 1:1 brain PE:PS, overlaid with chemical shifts predicted from (**a**) the crystal structure (circles) and (**b**) the relaxed crystal structure (crosses, PDB ID: 2QKS) [24]. The crystal structure was relaxed into a 1:1 PE:PS lipid bilayer with GROMACS, with chemical shifts for both structures predicted with SHIFTX2 [45,50]. The spectrum is indicative of a folded protein with abundant secondary structure.

As the DO construct was functional and produced the highest yield of all constructs, it was chosen to be uniformly isotopically labeled (^15^N, ^13^C) for further SSNMR analysis. In order to ascertain the quality of produced protein, chemical shifts were predicted using SHIFTX with and without MD relaxation of the X-ray coordinates into a bilayer composed of 1:1 PE:PS (PDB ID: 2QKS). Both sets of predicted chemical shifts show good agreement with experimental data, though not surprisingly the relaxed dataset more closely matches our SSNMR spectra (Figure 7 and Figure S9). Predicted chemical shifts from our MD relaxed structure suggest the experimental sample was homogenous throughout with regards to secondary and tertiary structure, and that there is good agreement between protein conformation in our own construct and the published crystal structure. In a broader context, this suggests that applications of codon harmonization are fully capable of producing properly folded protein.

### 3.6. Rare Codons in the Eukaryotic Gating Domain

**Figure 8:**
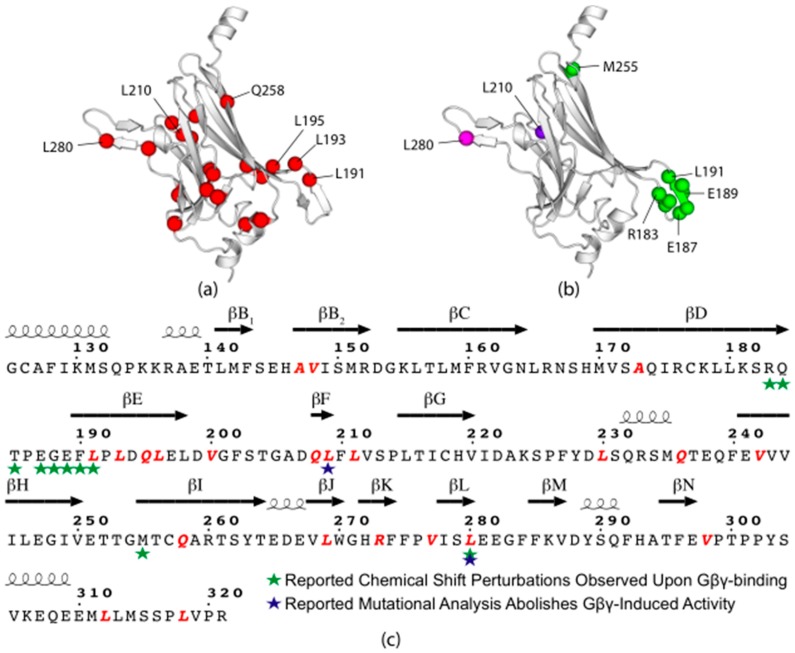
Chimera CTD rare codon analysis with crystal structure residue numbering (PDB ID: 2QKS) [24]. Compared are: (**a**) rare codons in red; (**b**) identified residues that undergo large CSPs (green) in response to binding, mutational analysis identified abolishment of channel activity (purple), or both (magenta); and (**c**) visualization of these residues as a function of primary sequence. Rare codons seem to correlate with or follow functional residues reported to be involved in Gβγ-binding. Formatting was adopted from the ESPRipt 3.0 output format (Figure S10) [34].

In GIRK channels, Gβγ is known to interact predominately with the CTD [25,60]. In the chimera CTD, rare codons seem to cluster near and within the βE, βF, and βJ-L strands (Figure S10). The crystal structure of GIRK2 (Kir3.2) in complex with Gβγ indicates interactions with the CTD at βK-N of one monomer’s CTD and βD and E of an adjacent monomer’s CTD [60]. Ethanol, another activating ligand, is also known to bind within the βD-E strands [61]. Chemical shift perturbations (CSPs) of residues R236-L244 within GIRK1′s βD-E strands, M308 in βI and L333 in βL (residues R183-L191, M255 and L280 within our chimera construct) in response to Gβγ-binding have been reported [62]. Mutational analysis has revealed His57, L262 and L333 are critical for activation, and mutations at these residues abolished Gβγ-induced GIRK1 activation [63,64]. Analysis reveals that the stretch in the βD-E sheets is followed by a cluster of rare codons, and the stand-alone residues either correspond to or are followed by immediate, downstream rare codons (Figure 8).

### 3.7. Rare Codons in the Prokaryotic Transmembrane Region

**Figure 9:**
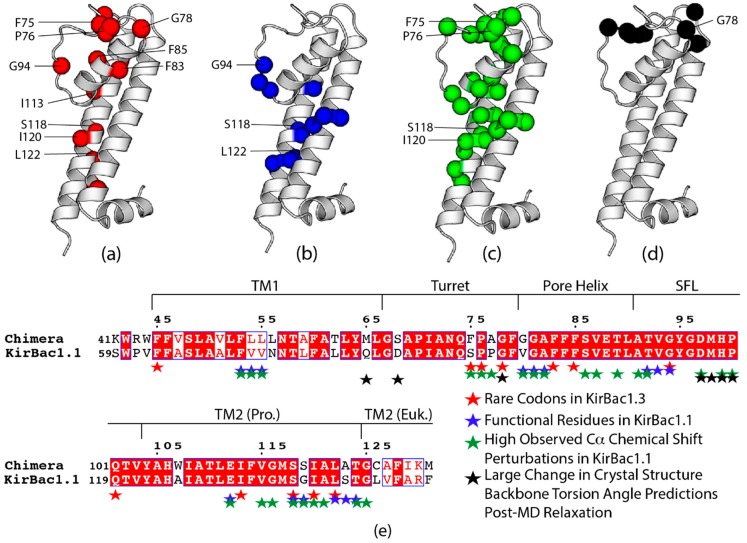
Visualization of chimera prokaryotic sequence analysis with crystal structure numbering conventions, comparing: (**a**) rare codons in red, labeled residues cluster in conformational hotspots; (**b**) functional residues in blue, labeled residues are rare codons; (**c**) chimera residues homologous to those with large observed CSPs in response to the inactive to active conformational transition, labeled residues are rare codons; (**d**) residues with large changes in predicted backbone torsion angles after Molecular Dynamics-based relaxation, labeled residues are rare codons; and (**e**) alignment of the prokaryotic region of the chimera with KirBac1.1, color-coded with observed residues in a-d (stars). In (**e**), solid red boxes indicate the same residue while red lettering with white background indicates similar residues monomeric structures in (**a**–**d**) were obtained from the crystal structure (PDB ID: 2QKS) prepared in PyMol and formatting in (**e**) was adopted from the ESPRipt 3.0 output format [24,34].

Our previous study over KirBac1.1 identified transmembrane residues with large CSPs in response to conformation exchange between the inactive and active states of the channel [65]. These residues are typically found near those known to be functional residues involved in conformational exchange within the transmembrane and extracellular regions and include both those observed in the inactive to active state transition and those found within alternate assignment pathways within the active state. Homologous residues within the Prokaryotic region of the chimera show some clustering with rare codons at the turret-pore helix interface and towards the base of TM2 (Figure 9a–c,e). Additionally, residues with large changes in predicted backbone torsion angles after MD relaxation show some overlap with the above homologous residues and rare codons at the turret-pore helix interface (Figure 9d–e, torsion angle predictions are available in Supplementary Information). Together, this suggests that functional regions within the transmembrane region, particularly the turret, pore helix, and the base of TM2 may contain rare codons in order for proper folding, and therefore functionality of these regions.

For example, the V98-G99-A100 triplet at the N-terminal of the pore helix in KirBac1.1 is implicated in pore helix rotation during conformational change. Homologous residues in the chimera (G80-G81-A82) are flanked by rare codons, one of which (G78) also experienced a large torsion angle prediction perturbation after MD-relaxation. Additionally, S136, G137, L140, S141, and T142 within TM2 of Kirbac1.1 are named ‘hinge residues’, so called because they are postulated to act as a pivot site for opening of the activation gate. Homologous residues in the chimera, S118, S119, L121, A122, and T123 are again clustered with or correspond to rare codons. Lastly, G94 in the selectivity filter is coded by a rare codon, though it is the only rare codon near or within the selectivity filter and therefore does not follow the same trend. That said, the selectivity filter is absolutely critical for proper K^+^ channel function and thus the presence of a rare codon within is intriguing.

## 4. Discussion

### 4.1. Translation Initiation and Elongation as a Two-Step Model of Protein Synthesis

Though codon harmonization of the coding sequences clearly has a positive effect on protein yield, there is variance across final yields of each construct. To this point, there has been little discussion as to why this may be the case. In light of mRNA secondary structure predictions, we suggest the observed experimental phenomena can be described by a simplified two-step model. Herein, a faster rate of initiation and a slower rate of elongation are postulated to produce the largest protein yield, not accounting for the myriad of other contributing factors.

First, initiation is the rate-limiting step and may be considered equivalent to the overall rate of translation, as has been previously described [55]. Therefore, the rate of initiation would dictate the overall amount of protein produced, with any mRNA 5′ secondary structure serving to reduce this value. This is in keeping with previous studies that have shown stronger 5′ secondary structure corresponds to less efficient initiation [66]. Second, application of codon harmonization serves to slow elongation in a site-directed manner such that structural ‘hotspots’ in the protein are allowed proper time to fold. The goal is to increase the fraction of protein produced that is properly folded and functional through harmonization, which is reflected in the decrease in overall predicted elongation efficiency (Table S1).

While the lack of mRNA 5′ secondary structure in the FO construct may lead to stronger initiation, the quick rate of elongation may not allow time for the protein to properly fold. This is due to the inclusion of mostly optimal codons, resulting in the lowest experimental yield. The NT and NT56 plasmids contain RBSs predicted to be occluded (Figure 5). These constructs represent an inverse of the FO construct: slower initiation and elongation, allowing time for the protein produced to properly fold but limiting the overall rate of translation. This may be the reason for increased yields over the FO construct, albeit not a drastic increase. The FT and DO constructs, like the FO, contain a predicted free RBS but have attenuated elongation due to the applied harmonization. Predictably, yields are increased over the FO construct. Though the MFE values for predicted 5′ secondary structures are somewhat similar, the difference may be enough to explain yield inequality (Figure 5). As mRNA secondary structure is known to inhibit translation, this is another control mechanism in conjunction with codon usage that can modulate quantity and quality of heterologously expressed proteins [67].

Here, we provide reasoning within a broader context for why this is the case, though this two-step model is perhaps only educated conjecture. For example, the observed yield inequalities may be due in part to translational stalling, premature termination, frameshifts and/or amino acid misincorporation [68]. Partial influence of any of these variables would serve to decrease the quality and quantity of the produced protein.

### 4.2. Other Variables as Co-Contributors to Yield Inequality

The transcription-translation system in prokaryotes is highly complicated. It is therefore probable that some other variables in concert with those discussed above are implicated in the observed experimental results. Here, we discuss three additional possibilities in some detail, though given the complexity it is again unlikely that a single factor is responsible for observed results.

In discussing coding sequence determinants of heterologous protein expression, we would be remiss in not discussing any role mRNA stability may play. mRNA stability refers to steady state processes that regulate mRNA half-life, or the rate of mRNA decay. It has previously been shown that codon usage plays a role in steady-state mRNA concentration, and that substitution of rare codons into a coding sequence negatively impacts mRNA stability [69,70]. It was also found that translation obstacles (e.g., initiation inhibition from 5′ mRNA secondary structure formation, ribosomal stalling, etc.) serve to decrease mRNA stability [69]. It is possible that mRNA stability plays a role in the discussed results, especially given the nature of the predicted 5′ secondary structure in the NT and NT56 constructs. To what extent is, however, unknown.

Contributions from purification may also play a role in the observed yield inequality. It is known that synonymous codon substitutions influence protein folding [11,57]. The prospect of misfolding has been addressed above. However, differences in folding across constructs may also affect accessibility of the C-terminal His-tag and therefore the efficiency of binding to the Ni^2+^ affinity column. This would, in turn, lead to under-estimation of tetrameric protein yield after down-stream purification steps. However, inaccessibility of the His-tag due to improper folding could not be transient in nature or else any contribution to yield inequality would be negligible. If such an interaction is non-transient, the His-tag would have to be buried within the CTD. This would likely very strongly influence gating and/or tetramerization of the channel. Such a state would be considered improperly or non-natively folded for the purposes of this work. As such, we do not believe that is the case with regards to these constructs. However, the mere possibility that changes in codon usage may serve to reveal or bury protein tags is an intriguing line of thought and should be considered when designing gene sequences.

A discussion of the effects of codon harmonization on protein solubility within the cell is also necessary. Unless an attempt is made to refold aggregated protein from inclusion bodies, insolubility of the target protein results in a ‘loss’ in recombinant yield. Proteins that find their way into the insoluble fraction are thought to do so as a function of misfolding events [71]. Furthermore, in vivo protein aggregation is linked to the rate of translation, with faster rates correlated to higher aggregation levels [72]. Translation rates are slower for rare codons but the effect on folding is not always positive and may depend on the specific codon used and the specific flanking codons (codon context) [73,74,75]. It is possible that differences in yields may be in part explained by over- or under-attenuation of the translational rate and substitution of rare codons into disadvantageous positions. In turn, this could lead to differences in solubility within the cell here, as membrane-inserted or aggregated within inclusion bodies. As the NT and NT56 sequences are relatively similar, it is likely that this issue would be shared between them and could explain why yields for these constructs were also similar. Codon harmonization aims to achieve the Goldilocks condition of translational modulation: not too fast, not too slow, but just right. Mismanagement of this rate can decrease or have no effect on yield. In this way, codon harmonization can be thought of as a technique to indirectly influence cellular solubility of recombinant proteins to increase the yield of the soluble, properly folded, and functional fraction.

### 4.3. Structural Significance of Rare Codons

In this chimera, rare codons in the eukaryotic portions are found to correspond to predominately hydrophobic residues (Figure S11a). Rare codons within the prokaryotic transmembrane region follow the same trend (Figure S11b). Due to the nature of rare codon locality, slower translation of these regions to allow for secondary structure formation is likely necessary to prevent hydrophobic residue exposure to solvent immediately after translation within the ribosome. Lack of or partial folding in these instances may contribute to heightened heterologous aggregation and sequestration in inclusion bodies. In the native organism this may be due to functional complexity, and therefore the necessity of precise folding into a conformation that will interact with gating ligands and channel K^+^ ions.

Within the CTD, the majority (14/20) of rare codons fall within β-sheet structures. This is inconsistent with the previous observation that β-sheets are depleted in conserved non-optimal codons, though discrepancies may arise across organisms [76]. The CTD is majority β-sheet, however. Therefore, the significance of rare codons within β-sheets in this instance is unknown. Given the distribution of rare codons in the native CTD, it could be possible that there is no correlation. That said, in the context of Gβγ-induced channel activation proper folding within the Kir3.2 CTD is likely crucial for the Gβγ docking event. Therefore, it is not outside the realm of possibility that rare codons within the β-sheets may still play a role in co-translational protein folding. This may serve to explain the observed patterns of CTD codon usage: inability of Gβγ to dock due to an improperly folded conformation would remove the main physiological gating partner in GIRK activation.

From a structural motif standpoint, rare codon usage in the transmembrane-extracellular region is consistent with previous observations that rare codons are more typically found at just before and within helices [76]. Though, there are a number of rare codons found midway within helices, which is outside the trends observed previously. This is again hypothesized to possibly slow translation and allow previous secondary elements to fold before translating the next motif, though the rules governing such are not well understood [15].

Given the noted correlations, rare codon usage may inform on functional and structural hotspots within Kir channels. However, the observed trends are not a perfect match. As such, experimental investigation is required to definitively gauge a correlation between rare codons and functional hotspots within the transmembrane and CTD regions of this construct.

### 4.4. Considerations in Recombinant Gene Design

Typically, it is assumed that when a gene is fully optimized by a vendor algorithm prior to purchasing, characteristics that have been implicated in reducing recombinant yield are taken into account and avoided. Though codon harmonization has been shown to increase yield up to 1000-fold, care must be taken when the end-user is responsible for gene construction [17]. It is not enough to simply apply harmonization for optimizing protein folding kinetics. In addition to mRNA 5′ secondary structure, tRNA depletion, SD-like sequences within the mRNA, codon repeats that lead to ribosomal stalling and aborted translation, ribosomal jamming, mRNA stability, and other variables must be taken into account [15]. A likely reason codon harmonization does not see more widespread use is that it is very difficult to control for all contributing variables, and therefore application increases yield in only some cases. Therefore, we suggest that the method of application is arbitrary. Use of Codon Wizard, DUMB optimization, or any other harmonization platform over another likely does not matter, and therefore the observed yield differences in the NT and NT56 constructs cannot be blamed on the Codon Wizard harmonization methodology itself. Instead, we find it far more likely that the culprits are sequence and structural motifs in the mRNA that, to our knowledge, harmonization methods do not typically take into account.

### 4.5. Codon Harmonization as an Easy-to-Implement Technique with Applications in Structural Studies

Despite aforementioned pitfalls, codon harmonization is worth investigating as a technique for structural study optimization. In all cases, harmonization of the plasmid produced yields greater than the traditional FO sequence though not statistically significantly in all cases. The goal of this experiment was to gauge the efficacy of different approaches by which codon harmonization can be applied to increase yields of difficult-to-produce membrane proteins. The observed yield inequality serves to highlight the need for continued experimentation with different approaches in order to fine-tune harmonization application. Structural studies of membrane proteins can often be cost-prohibitive, requiring expensive detergents and, in the case of NMR, costly isotopes. Even a roughly two-fold yield increase, as shown, can cut operational costs.

## 5. Conclusions

In summary, we apply systematic deoptimization in the coding sequence of a chimeric K^+^ channel based on the native codon usage, known as codon harmonization, to increase recombinant protein yield. This increase was not equal, and reasoning was supplied for why this was the case. Efflux assay results showed all constructs were biologically functional. The DO construct, with a nearly two-fold yield increase, was selected to for NMR studies as the sample depicting properly folded protein with abundant secondary structure. C-C 2D correlation spectroscopy compared to chemical shifts predicted from the crystal structures suggests the protein produced is properly folded. This experiment has wider implications for the structural biology community, as application can increase protein production efficiency, which is of importance to anyone attempting to perform structural studies of difficult-to-produce proteins, such as membrane proteins.

## Supplementary Materials

The following are available online at https://www.mdpi.com/2218-273X/10/3/430/s1, Figure S1: Native Codon Usage, Figure S2: Full-length FO Construct Relative Codon Usage, Figure S3: Full-length DO Construct Relative Codon Usage, Figure S4: Full-length NT Construct Relative Codon Usage, Figure S5: Full-length NT56 Construct Relative Codon Usage, Figure S6: Full-length FT Construct Relative Codon Usage, Figure S7: Heat Maps of RCU; Figure S8: mRNA Characteristics Plotted vs. Construct Yield; Figure S9: ^13^C-^13^C Correlation Spectrum with Predicted Crystal Structure Chemical Shifts, Figure S10: Chimera-Kir3.2 CTD Alignment, Figure S11: Codon Usage Distribution by Residue Type, Table S1: mRNA Characteristics by Construct, Spreadsheet S1: Crystal Structure-Predicted Backbone Torsion Angles.
