# Supplementary material for: Codon Harmonization of a Kir3.1-KirBac1.3 Chimera for Structural Study Optimization
Source: Biomolecules. 2020 Mar 10;10(3):430. doi: 10.3390/biom10030430 (PMC7175280; doi:10.3390/biom10030430)
Supplement: Supplementary file 1 [file biomolecules-10-00430-s001.zip › To Be Packed Up 2/Codon Harmonization SI.docx]

*Supporting information for*

**Codon Harmonization of a Kir3.1-Kirbac1.3 Chimera for Structural Study Optimization**

Evan van Aalst^1, #^, Maryam Yekefallah^1, #^, Anil K. Mehta^2^, Isaac Eason^1^, and Benjamin Wylie ^1,^ *

^1^	Department of Chemistry and Biochemistry, Texas Tech University, Lubbock, Texas 79423, USA

^2^	National High Magnetic Field Laboratory and McKnight Brain Institute, University of Florida, Box 10015, Gainesville, FL 32610, USA

*****	Correspondence: [benjamin.j.wylie@ttu.edu](mailto:benjamin.j.wylie@ttu.edu)

**#**	Authors contributed equally to the work in this paper

Experimental Section

*Gene Constructs*

Fully Optimized (FO):

5’ – CC***ATG***GCAACCAAACCGACCGATCGTCCGAGCGCATTTGGTAATGGTGAAGC

ACGTGCACCGACACGTCGTCTGGTTCCGCGTGGTAGCAAAAAACGTCAGCGTTTTGTTGATAAAAATGGTCGTTGCAATGTGCAGCATGGTAATCTGGGTAGCGAAACCAGCCGTTATCTGAGCGACCTGTTTACCACACTGGTTGATCTGAAATGGCGTTGGTTTTTTGTTAGCCTGGCAGTTCTGTTTCTGCTGCTGAATACCGCATTTGCAACCCTGTATATGCTGGGTAGTGCACCGATTGCAAATCAGTTTCCGGCAGGTTTTGGTGGTGCATTTTTCTTTAGCGTTGAAACCCTGGCAACCGTTGGTTATGGTGATATGCATCCGCAGACCGTTTATGCACATTGGATTGCGACCCTGGAAATTTTTGTTGGTATGAGCAGCATTGCACTGGCCACCGGTTGTGCATTTATCAAAATGAGCCAGCCGAAAAAACGTGCAGAAACCCTGATGTTTAGCGAACATGCAGTTATTAGCATGCGTGATGGTAAACTGACACTGATGTTTCGTGTGGGTAATCTGCGTAATAGCCATATGGTTAGCGCACAGATTCGTTGTAAACTGCTGAAAAGCCGTCAGACACCGGAAGGTGAATTTCTGCCGCTGGATCAGCTGGAACTGGATGTTGGTTTTAGTACCGGTGCAGATCAGCTGTTTCTGGTTAGTCCGCTGACCATTTGTCATGTTATTGATGCAAAAAGCCCGTTCTATGATCTGAGCCAGCGTAGCATGCAGACCGAACAGTTTGAAGTTGTTGTTATTCTGGAAGGCATTGTTGAAACCACCGGTATGACCTGTCAGGCACGTACCAGCTATACCGAAGATGAAGTTCTGTGGGGTCATCGTTTTTTTCCGGTTATTAGCCTGGAAGAGGGTTTCTTCAAAGTTGATTATAGCCAGTTTCACGCCACCTTTGAAGTTCCGACACCGCCTTATAGCGTTAAAGAACAAGAAGAAATGCTGCTGATGAGCAGTCCGCTGGTGCCTCGTGGTAGTGGTGGTCTGGAAGGATCC – 3’

DUMB Optimized (DO):

5’ – CC***ATG***GCAACCAAACCGACCGATCGTCCGAGCGCATTTGGTAATGGTGAAGC

ACGTGCACCGACACGTCGTCTGGTTCCGCGTGGTAGCAAAAAACGCCAGCGCTTTGTTGATAAAAATGGTCGCTGCAATGTGCAGCATGGAAATCTGGGTAGCGAAACCTCCCGCTATTTGTCGGATTTATTTACGACCCTGGTTGATTTAAAATGGAGATGGTTCTTTGTGAGCCTGGCAGTTCTGTTTCTGCTGCTGAATACGGCATTTGCTACCCTGTATATGCTGGGTTCCGCACCGATTGCTAATCAGTTCCCCGCTGGGTTTGGTGGTGCATTCTTTTTCTCCGTGGAAACCCTGGCTACCGTGGGGTATGGTGATATGCATCCGCAAACGGTGTATGCACATTGGATTGCTACCCTGGAAATATTTGTGGGTATGTCGTCCATAGCACTCGCCACCGGTTGTGCATTTATTAAAATGAGTCAGCCGAAAAAACGCGCAGAAACCTTAATGTTTAGCGAACACGCTGTAATTAGTATGCGTGATGGCAAATTAACGTTAATGTTTCGCGTGGGTAATCTGCGCAATAGCCATATGGTAAGTGCTCAAATTCGCTGTAAACTGTTAAAATCTCGCCAGACACCGGAAGGAGAATTTTTGCCGTTGGATCAATTGGAACTGGATGTAGGGTTTTCCACAGGCGCAGATCAATTGTTTTTGGTTAGTCCGTTAACAATCTGTCATGTTATTGATGCAAAAAGCCCGTTTTATGATCTCAGCCAGCGGAGCATGCAAACGGAACAGTTTGAAGTAGTAGTAATTCTGGAAGGCATTGTTGAAACCACAGGCATGACGTGCCAAGCTCGGACATCGTATACAGAAGATGAAGTTTTGTGGGGGCATAGATTTTTTCCGGTAATCTCTCTAGAAGAGGGCTTTTTTAAAGTAGATTATAGCCAGTTTCACGCTACCTTTGAAGTACCGACACCGCCGTATAGCGTTAAAGAACAAGAAGAAATGTTGTTAATGTCAAGTCCGCTAGTGCCTCGTGGTAGTGGTGGTCTGGAAGGATCC – 3’

Codon Wizard No Tolerance (NT):

5’ -CC***ATG***GCTACTAAGCCTACCGATCGGCCCTCGGCATTTGGTAACGGTGAAGCT

CGTGCACCCACCCGTCGTCTGGTTCCGCGTGGTAGCAAAAAGCGGCAGCGGTTTGTTGATAAAAACGGCCGGTGTAACGTTCAGCATGGGAACCTGGGCAGCGAAACCTCTCGGTACTTGTCGGATTTATTTACGACCCTGGTTGACTTAAAATGGCGATGGTTCTTTGTTAGCCTGGCAGTTCTGTTTCTGCTGCTGAACACCGCATTTGCAACCCTGTACATGCTGGGTAGCGCACCGATTGCAAACCAGTTCCCTGCAGGGTTTGGTGGTGCATTCTTTTTCAGCGTTGAAACCCTGGCAACCGTTGGGTACGGTGATATGCATCCGCAAACCGTTTACGCACATTGGATTGCAACCCTGGAAATATTTGTTGGTATGTCGAGCATAGCATTGGCAACCGGCTGTGCTTTTATCAAAATGAGCCAGCCGAAGAAACGGGCAGAAACCTTAATGTTCAGCGAACATGCTGTAATCAGCATGCGCGATGGAAAGTTAACGTTAATGTTTCGGGTTGGCAACCTGCGGAACAGCCATATGGTCAGCGCTCAGATCCGGTGTAAACTGTTAAAGAGCCGGCAGACACCGGAAGGGGAATTTTTGCCGTTGGATCAATTGGAGCTGGACGTAGGGTTCTCTACAGGAGCAGACCAATTGTTCTTGGTTAGCCCGTTAACAATATGTCATGTTATCGACGCAAAGAGCCCGTTTTACGATCTCAGCCAGAGAAGCATGCAAACGGAGCAGTTTGAAGTAGTCGTCATCCTGGAGGGCATAGTTGAGACCACAGGAATGACGTGTCAAGCGAGAACATCTTACACAGAAGATGAGGTTTTGTGGGGGCATCGATTCTTTCCGGTAATAAGCCTAGAGGAGGGATTTTTCAAGGTCGACTACAGCCAGTTTCATGCTACCTTCGAGGTACCGACCCCGCCGTACAGCGTTAAGGAACAGGAAGAGATGTTGTTAATGAGCAGCCCGCTGGTGCCTCGTGGTAGTGGTGGTCTGGAAGGATCC – 3’

Codon Wizard No Tolerance, 56% G:C (NT56):

5’ – CC***ATG***GCTACGAAGCCTACAGACCGGCCATCTGCATTCGGCAACGGTGAGGC

TAGAGCACCAACCCGCAGACTGGTTCCGCGTGGTAGCAAGAAACGGCAAAGATTTGTGGACAAAAACGGTCGGTGCAACGTGCAGCATGGGAACCTGGGCAGTGAAACCTCTAGATATTTATCGGACCTCTTTACGACCCTGGTTGACCTCAAGTGGCGGTGGTTTTTTGTTAGCCTGGCCGTGCTGTTTCTGTTACTGAACACCGCATTCGCCACCTTATATATGTTAGGTAGCGCACCGATTGCAAATCAATTCCCTGCCGGATTCGGCGGCGCCTTCTTTTTCAGCGTGGAGACCCTGGCCACCGTGGGATACGGTGACATGCACCCGCAGACAGTGTACGCCCACTGGATCGCCACATTAGAAATATTCGTGGGCATGTCGAGCATCGCCTTAGCAACAGGCTGCGCTTTCATTAAGATGAGCCAGCCGAAGAAGCGGGCCGAGACCCTCATGTTCAGTGAGCACGCGGTCATCAGCATGAGAGACGGCAAGTTAACTCTCATGTTCCGGGTGGGCAACCTGAGAAACAGTCACATGGTCAGTGCGCAGATTCGGTGCAAGCTGTTAAAGAGTCGGCAGACACCGGAGGGGGAGTTCCTCCCGCTCGACCAACTCGAGCTGGACGTCGGGTTTTCTACAGGGGCCGATCAGCTCTTCCTCGTGAGCCCGCTCACGATCTGCCACGTGATCGACGCCAAGAGCCCTTTCTACGATCTCAGTCAGCGGAGCATGCAGACGGAGCAGTTTGAGGTCGTCGTAATCCTGGAGGGTATAGTGGAGACCACGGGCATGACGTGCCAGGCTAGAACGTCTTACACGGAAGACGAGGTGCTCTGGGGGCACCGGTTTTTCCCGGTAATCAGTCTCGAGGAAGGCTTCTTCAAGGTCGACTACAGCCAATTCCACGCGACCTTCGAGGTCCCGACCCCGCCTTACAGCGTTAAGGAGCAGGAAGAGATGTTACTCATGAGCAGCCCGCTGGTGCCTCGTGGTAGTGGTGGTCTGGAAGGATCC – 3’

Codon Wizard Full Tolerance (FT):

5’ – CC***ATG***GCAACTAAACCCACTGATCGGCCCTCTGCGTTTGGGAACGGGGAAGC

CAGGGCTCCTACCCGTAGACTGGTTCCGCGTGGTAGCAAAAAGAGGCAGAGGTTTGTAGACAAAAACGGGAGGTGTAACGTCCAGCATGGCAACCTCGGATCTGAAACGTCGCGTTATTTAAGTGATCTCTTTACCACTCTCGTCGACTTGAAATGGCGTTGGTTCTTTGTATCTTTGGCTGTTTTGTTTCTTCTTCTCAATACTGCGTTTGCGACCTTGTATATGCTGGGATCTGCTCCGATAGCAAACCAGTTCCCTGCTGGGTTTGGAGGGGCTTTTTTTTTTTCGGTCGAAACTCTTGCTACCGTTGGTTACGGGGATATGCATCCCCAAACGGTATATGCACATTGGATTGCTACGCTGGAGATATTTGTAGGGATGAGCTCGATAGCATTGGCTACTGGCTGTGCTTTTATCAAAATGAGCCAGCCCAAGAAGCGTGCGGAAACTTTAATGTTCTCTGAGCATGCTGTGATCTCCATGCGCGATGGAAAGCTGACGCTGATGTTTCGGGTTGGGAACCTGCGTAACTCTCATATGGTTTCGGCTCAAATCCGCTGTAAACTACTGAAGTCCCGACAAACCCCCGAAGGTGAATTTCTGCCGCTGGATCAATTGGAGCTCGATGTGGGCTTTAGCACAGGCGCAGACCAACTGTTCTTGGTCAGCCCGTTGACAATATGCCACGTCATAGACGCTAAGTCACCATTCTATGATTTAAGCCAGCGTTCGATGCAGACGGAACAGTTCGAGGTGGTCGTGATACTCGAAGGAATAGTTGAGACTACCGGTATGACATGCCAAGCCAGAACATCGTATACCGAAGACGAAGTCCTGTGGGGGCATCGCTTCTTTCCCGTTATTAGCTTAGAGGAAGGATTTTTCAAGGTCGACTACTCGCAATTTCATGCTACTTTCGAAGTGCCAACTCCCCCGTACTCAGTAAAGGAACAAGAAGAAATGCTATTGATGTCTTCACCGCTGGTGCCTCGTGGTAGTGGTGGTCTGGAAGGATCC – 3’

*Primers*

pQE-60 Fwd Primer: GTA TCA CGA GGC CCT TTC GTC T

pQE-60 Rev Primer: CAT TAC TGG ATC TAT CAA CAG GAG

*Minimal Media Recipes*

Solution C, 1 L: adjust pH to 6.7 with KOH

7.3 g KOH

50 ml Metal 44

10 g Nitrilotriacetic acid

24 g MgCl_2_·6H_2_O

3.335 g CaCl_2_·2H_2_O

Metal 44 solution, 100 mL: Stored in a dark glass bottle at 4 °C

0.327 g K_2_EDTA·2H_2_O

0.522 g ZnCl_2_

0.502 g FeCl_2_·4H_2_O

0.18 g MnCl_2_·4H_2_O

0.0185 g (NH_4_)_6_Mo_7_O_24_·6H_2_O

0.0156 g CuCl_2_·2H_2_O

0.0248 g Co(NO_3_)_2_·6H_2_O

0.0114 g Boric Acid

**Figure S1.** Native codon usage for Kirbac1.3 (residues 1-29, 72-151) and Kir3.1 (residues 30-71, 152-345) mapped onto the corresponding full-length Chimera residues. Numbers in parentheses correspond to the crystal structure numbering convention. Adopted from GCUA output format [1].

**Figure S1.** Native codon usage for Kirbac1.3 (residues 1-29, 72-151) and Kir3.1 (residues 30-71, 152-345) mapped onto the corresponding full-length Chimera residues. Numbers in parentheses correspond to the crystal structure numbering convention. Adopted from GCUA output format [1].

**Figure S2**. Full-length FO Construct Relative Codon Usage. Numbers in parentheses correspond to the crystal structure numbering convention. Adopted from GCUA output format [1].

**Figure S2**. Full-length FO Construct Relative Codon Usage. Numbers in parentheses correspond to the crystal structure numbering convention. Adopted from GCUA output format [1].

**Figure S3.** Full-length DO Construct Relative Codon Usage. Numbers in parentheses correspond to the crystal structure numbering convention. Adopted from GCUA output format [1].

**Figure S3.** Full-length DO Construct Relative Codon Usage. Numbers in parentheses correspond to the crystal structure numbering convention. Adopted from GCUA output format [1].

**Figure S4.** Full-length NT Construct Relative Codon Usage. Numbers in parentheses correspond to the crystal structure numbering convention. Adopted from GCUA output format [1].

**Figure S4.** Full-length NT Construct Relative Codon Usage. Numbers in parentheses correspond to the crystal structure numbering convention. Adopted from GCUA output format [1].

**Figure S5.** Full-length NT56 Construct Relative Codon Usage. Numbers in parentheses correspond to the crystal structure numbering convention. Adopted from GCUA output format [1].

**Figure S5.** Full-length NT56 Construct Relative Codon Usage. Numbers in parentheses correspond to the crystal structure numbering convention. Adopted from GCUA output format [1].

**Figure S6.** Full-length FT Construct Relative Codon Usage. Numbers in parentheses correspond to the crystal structure numbering convention. Adopted from GCUA output format [1].

**Figure S6.** Full-length FT Construct Relative Codon Usage. Numbers in parentheses correspond to the crystal structure numbering convention. Adopted from GCUA output format [1].

**Figure S7.** Relative codon usage GCUA output values per codon mapped onto the Chimera crystal structure monomer (PDB ID: 2QKS), red in: (a) native; (b) FO; (c) DO; (d) NT; (e) NT56; (f) FT; shows rarer codons [1,2].

**Figure S8.** Construct yield plotted against values listed in Table S1: (a) CAI; (b) N_c_; (c) EE; (d) ARCU. Non-linear correlations suggest interplay between these and potentially uninvestigated characteristics.

**Figure S9.** ^13^C-^13^C DARR correlation spectrum of the GIRK chimera reconstituted into 1:1 brain PE:PS, overlaid with chemical shifts predicted from (a) the crystal structure (circles) and (b) the relaxed crystal structure (crosses). The crystal structure was relaxed into a 1:1 PE:PS lipid bilayer with GROMACS, with chemical shifts for both structures predicted with SHIFTX2 [3,4].

**Figure S10.** Chimera alignment to Kir3.2 CTD. Solid red indicates the same residue. Red lettering with white background indicates similar residues. Adopted from ESPRit output format [5].

**Figure S11.** Codon usage distribution by residue type in the (a) Eukaryotic and (b) Prokaryotic portions of the Chimera.

**Table S1.** mRNA characteristics by construct.

|  | Codon Adaptivity Index (% of max) [6] | Effective Number of Codons [7] | Elongation Efficienct (% of max) [8] | Average Relative Codon Usage (% of max) |
| --- | --- | --- | --- | --- |
| Fully Optimized Construct | 88.0 | 33.2 | 63.4 | 98.11 |
| DUMB Optimized Construct | 79.1 | 43.7 | 51.5 | 88.20 |
| No Tolerance Construct | 70.7 | 45.7 | 53.7 | 81.75 |
| No Tolerance 56% G:C Construct | 67.0 | 48.4 | 49.1 | 76.20 |
| Full Tolerance Construct | 65.3 | 55.4 | 46.7 | 77.09 |

**References:**

1.	Fuhrmann, M.; Hausherr, A.; Ferbitz, L.; Schodl, T.; Heitzer, M.; Hegemann, P. Monitoring dynamic expression of nuclear genes in Chlamydomonas reinhardtii by using a synthetic luciferase reporter gene. *Plant Mol Biol* **2004**, *55*, 869-881, doi:10.1007/s11103-004-2150-6.

2.	Nishida, M.; Cadene, M.; Chait, B.T.; MacKinnon, R. Crystal structure of a Kir3.1-prokaryotic Kir channel chimera. *Embo J* **2007**, *26*, 4005-4015, doi:10.1038/sj.emboj.7601828.

3.	Abraham, M.J.; Murtola, T.; Schulz, R.; Páll, S.; Smith, J.C.; Hess, B.; Lindahl, E. GROMACS: High performance molecular simulations through multi-level parallelism from laptops to supercomputers. *SoftwareX* **2015**, *1-2*, 19-25, doi:10.1016/j.softx.2015.06.001.

4.	Han, B.; Liu, Y.; Ginzinger, S.W.; Wishart, D.S. SHIFTX2: significantly improved protein chemical shift prediction. *J Biomol NMR* **2011**, *50*, 43-57, doi:10.1007/s10858-011-9478-4.

5.	Robert, X.; Gouet, P. Deciphering key features in protein structures with the new ENDscript server. *Nucleic Acids Research* **2014**, *42*, W320-W324, doi:10.1093/nar/gku316.

6.	Sharp, P.M.; Li, W.H. The codon Adaptation Index--a measure of directional synonymous codon usage bias, and its potential applications. *Nucleic Acids Res* **1987**, *15*, 1281-1295, doi:10.1093/nar/15.3.1281.

7.	Wright, F. The 'effective number of codons' used in a gene. *Gene* **1990**, *87*, 23-29, doi:10.1016/0378-1119(90)90491-9.

8.	Xia, X. DAMBE7: New and Improved Tools for Data Analysis in Molecular Biology and Evolution. *Mol Biol Evol* **2018**, *35*, 1550-1552, doi:10.1093/molbev/msy073.
